# 7T MRI and Computational Modeling Supports a Critical Role of Lead Location in Determining Outcomes for Deep Brain Stimulation: A Case Report

**DOI:** 10.3389/fnhum.2021.631778

**Published:** 2021-02-11

**Authors:** Lauren E. Schrock, Remi Patriat, Mojgan Goftari, Jiwon Kim, Matthew D. Johnson, Noam Harel, Jerrold L. Vitek

**Affiliations:** ^1^Department of Neurology, University of Minnesota, Minneapolis, MN, United States; ^2^Center for Magnetic Resonance Research, Department of Radiology, University of Minnesota, Minneapolis, MN, United States; ^3^Department of Biomedical Engineering, University of Minnesota, Minneapolis, MN, United States; ^4^Department of Chemical Engineering and Materials Science, University of Minnesota, Minneapolis, MN, United States

**Keywords:** deep brain stimulation, subthalamic nucleus, Parkinson’s disease, electrode location, ultra-high field MRI, computational modeling, case report

## Abstract

Subthalamic nucleus (STN) deep brain stimulation (DBS) is an established therapy for Parkinson’s disease motor symptoms. The ideal site for implantation within STN, however, remains controversial. While many argue that placement of a DBS lead within the sensorimotor territory of the STN yields better motor outcomes, others report similar effects with leads placed in the associative or motor territory of the STN, while still others assert that placing a DBS lead “anywhere within a 6-mm-diameter cylinder centered at the presumed middle of the STN (based on stereotactic atlas coordinates) produces similar clinical efficacy.” These discrepancies likely result from methodological differences including targeting preferences, imaging acquisition and the use of brain atlases that do not account for patient-specific anatomic variability. We present a first-in-kind within-patient demonstration of severe mood side effects and minimal motor improvement in a Parkinson’s disease patient following placement of a DBS lead in the limbic/associative territory of the STN who experienced marked improvement in motor benefit and resolution of mood side effects following repositioning the lead within the STN sensorimotor territory. 7 Tesla (7 T) magnetic resonance imaging (MRI) data were used to generate a patient-specific anatomical model of the STN with parcellation into distinct functional territories and computational modeling to assess the relative degree of activation of motor, associative and limbic territories.

## Introduction

Deep brain stimulation (DBS) in the subthalamic nucleus (STN) is an effective therapy for the motor symptoms associated with Parkinson’s disease (PD). Although STN DBS has been performed for PD for more than 30 years, the optimal site for implantation within the target remains under debate. In fact, in a recent survey thirty-three movement disorders specialists were presented with the same canonical representation of the STN and were asked to indicate their preferred targeting location. While results showed that there was some clustering for the preferred target observed in the dorsolateral STN and subthalamic area, the suggested targets were heterogeneous, and no consensus existed. The authors concluded that the optimal target for STN DBS needed further verification (Hamel et al., 2017). Furthermore, while many have argued that the lead should be placed within the sensorimotor territory of the STN (Herzog et al., 2004; Wodarg et al., 2012; Horn et al., 2017), others report similar effects with leads placed in the associative or motor territory of the STN (Welter et al., 2014), and some assert that placing a DBS lead “anywhere within a 6 mm diameter cylinder centered in the presumed middle of the STN based on stereotactic atlas coordinates produces similar clinical efficacy” (McClelland et al., 2005). Still others have argued that the best location includes a region just dorsal to the STN (Plaha et al., 2006; Kasasbeh et al., 2013). These discrepancies likely contribute to the significant variability of clinical outcomes observed in clinical trials and day-to-day DBS therapy across centers (Deuschl et al., 2006; Follett et al., 2010; Vitek et al., 2020) as well as the unexpectedly high rate of documented DBS lead revisions (Rolston et al., 2016). Possible causes for these discrepancies and clinical observations include targeting preferences, image quality and the use of brain atlases that do not account for patient-specific anatomic variability.

Anatomical and imaging studies have divided the STN into three separate, though partially overlapping zones, serving motor, associative, and limbic function (Lambert et al., 2012; Haynes and Haber, 2013). 7 Tesla (7T) MRI techniques combined with post-processing analysis of diffusion weighted images have provided compelling evidence that the distribution pattern of these functional zones may be patient-specific (Plantinga et al., 2018).

Here, we take advantage of these patient-specific 7T MRI techniques as well as computational modeling of pathway activation to seize a unique opportunity to study a patient who developed severe, reversible depression after undergoing STN DBS with a lead placed in the associative/limbic territories. The patient subsequently required revision of lead location, which alleviated the mood side effects and improved motor function. We determined the location of individual stimulating contacts within the subterritories of the STN following the 1st and 2nd implantations. We then constructed a patient-specific computational model of the STN DBS settings to quantitatively estimate the degree of activation of specific neuronal pathways that were modulated at each clinical stimulation setting. Stimulation site within the STN was found to be a crucial determinant of this patient’s motor outcomes and presence or absence of mood side effects, consistent with the hypothesis that DBS outcomes are critically dependent on lead location.

## Case Presentation

A 52-year-old patient with a 14-year history of PD underwent bilateral STN DBS for treatment of motor fluctuations with severe rigidity and bradykinesia during off periods and frequent disabling dyskinesias when on, despite optimization of anti-parkinsonian medications. The patient reported longstanding baseline anxiety but had never required psychiatric treatment. There was no history of a mood disorder.

The patient was a participant in a clinical trial of STN DBS for the treatment of PD. The study was approved by the University of Minnesota’s IRB board and the patient provided informed consent (University of Minnesota Twin Cities, MN, United States, RRID:SCR_011674).

With the patient awake, single unit microelectrode mapping was performed to define the borders and sensorimotor territory of the STN through identification of cells whose discharge was modulated by passive movements of the contralateral limbs. This was followed by intraoperative test stimulation from the DBS lead (BSC-DB-2201, Boston Scientific) to assess side effect thresholds. The left STN lead was implanted first. Test stimulation with the DBS lead at 130 Hz, 90 μs using contacts 2−/4+ elicited paresthesia at 4.0 mA, while tongue contractions were elicited using contacts 4−/2+ at 6 mA. Following implantation of the left STN, the right STN was mapped and the lead implanted. During test stimulation, however, the threshold for left face and chest paresthesia was unacceptably low (2.0 mA), suggesting proximity to medial lemniscal fibers running posteromedial to the STN. Thus, the lead was repositioned 2 mm anteriorly. Test stimulation at this location revealed transient paresthesia at 5.0 mA.

Shortly after programming the second (right) side (see Table 1 for programming settings) the patient became hypomanic and severely anxious, requiring an urgent clinic appointment. The DBS settings were adjusted with reduction of the stimulation amplitude bilaterally and switching the right lead to activation of a more dorsal contact, following a strategy previously outlined (Greenhouse et al., 2011). On these settings, and despite a further dorsal shift of stimulation on the right lead, the patient developed severe depression, anxiety, and frequent crying. Four months after initial implantation, the patient attempted suicide with a pain medication overdose; the patient recovered without medical treatment. DBS was turned off on both sides at this time and the patient was hospitalized briefly for psychiatric treatment. Her mood significantly improved with DBS turned off.

**TABLE 1:** DBS programming settings and UPDRS III Motor Scores.

	Pre-surgical baseline		Initial settings (DBS is turned ON)	DBS with misplaced R STN lead on Low settings (due to mood side effects)	Settings at time of suicide attempt and formal mood testing	Pre-surgical baseline (Revision surgery)	DBS with revised R STN lead at optimized settings
Time from initial lead implantation	(−)4 weeks		(+)4 weeks	(+)16 weeks	(+)24 weeks	(+) 30 weeks	(+)3 years; (+)2 years from R STN revision
**Left DBS lead**	NA		Case+, 3−; 1.5 mA; 60 μs; 130 Hz	Case+, 3−; 0.9 mA; 60 μs; 130 Hz	Case+, 4−; 0.6 mA; 60 μs; 130 Hz	NA	Case+, 4−; 1.2 mA; 60 μs; 130 Hz
**Right DBS lead**	NA		Case+, 11−; 1.3 mA; 60 μs; 130 Hz	Case+, 12− (70%), 13− (30%); 0.6 mA; 60 μs; 130 Hz	Case+, 12− (70%), 13− (30%); 1.0 mA; 60 μs; 130 Hz	NA	Revised lead: Case+, 12−; 1.2 mA; 60 μs; 130 Hz
**Medication state**	OFF meds	ON meds	OFF meds	OFF meds	ON meds	OFF meds	OFF meds
**UPDRS III LEFT body subscore** (% improvement from pre-op baseline)	13	6	13 (0%; scored with DBS still OFF)	9 (30.77%)	NA	13	1 (92.31%)
**UPDRS III RIGHT body subscore** (% improvement from pre-op baseline)	17	7	14 (17%; scored with DBS still OFF)	8 (52.94%)	NA	14	2 (88.24%)
**UPDRS III subscore** (% improvement from pre-op baseline)	48	17	45 (6.25%; scored with DBS still OFF)	31 (35.42%)	NA	49	14 (70.83% compared to pre-DBS; 71.43% compared to pre-revision baseline)

*DBS, Deep Brain stimulation; C+, Case as anode; 3−, Contact 3 as cathode; 12− (70%), Contact 12 as cathode receiving 70% of the current; UPDRS III denotes Unified Parkinson Disease Rating Scale Part 3 (motor subsection). The thick vertical line indicates the timepoint for DBS implantations.*

The patient returned to clinic for formal assessment of stimulation effects on mood (see Table 2). Initial assessment with DBS OFF revealed the patient was euthymic with no depression, anxiety, or hypomania. For the subsequent evaluations the patient was blinded to the stimulation state. 2.5 h after taking morning medications, the right DBS lead was first activated to the settings the patient was on at the time of the suicide attempt. The left lead, which had not been associated with any mood changes when ON, remained OFF. Within 2 min of stimulation onset the patient reported feeling “a profound sadness, hopelessness, despair, and loss of trust… I don’t think I can make it.” At 3 min the patient started crying and the spouse observed “a noticeable change in her eyes, as if she is no longer my wife.” At 4 min the negative mood effects seemed to peak, and the patient reported feeling “alone. I feel like I’m pulling away. It’s hard to see things will ever get better.” DBS was then turned OFF without notifying the patient, and immediately the patient said “I feel hopeful. The room just became brighter.” Within 6 min of turning off the stimulation her mood had returned to baseline.

**TABLE 2:** Formal assessment of mood effects from DBS.

DBS state (ON medication)	DBS settings	Mood assessment
DBS OFF	NA	Euthymic. No depression, anxiety, or hypomania.
Right DBS lead ON; Left DBS lead OFF	C+;12− (60%);13− (40%);1.0 mA;60 μs;130 Hz	2 min: Patient felt the onset of a profound sadness, sense of hopelessness, despair, and loss of trust. 3 min: Patient started to cry; husband reported a clear change in her eyes. 4 min: Depressed mood became overwhelming: “I feel like I’m pulling away. I feel alone. I don’t see that things will ever get better. I don’t think I could make it.” DBS turned back OFF without warning: Immediately the patient reported “I feel more hopeful. The room seems brighter.” 5 min after DBS turned OFF: Mood reported to be 80% back to baseline.
Right DBS lead OFF; Left DBS lead ON	C+;4−;0.6 mA;60 μs;130 Hz	3 min: Mild increase in right-sided dyskinesia. Mood unchanged, euthymic. No changes in mood during 10 min of monitored stimulation.

*DBS, Deep Brain Stimulation; NA, Not Applicable; Min, minutes.*

**FIGURE 1:**
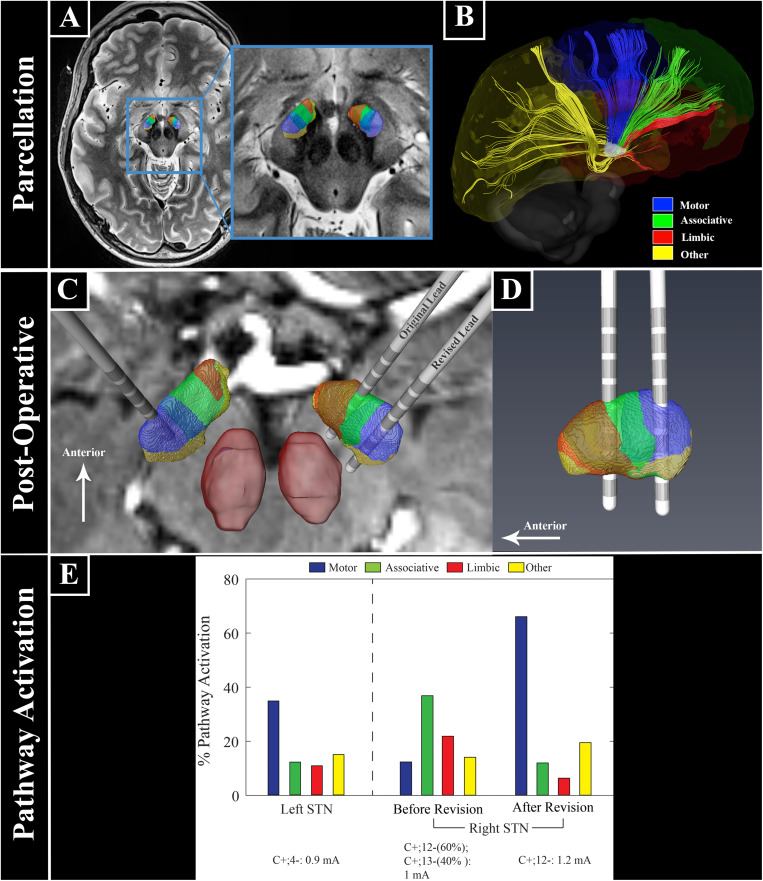
Ultra high field 7 Tesla MR images for patient-specific STN parcellation, lead location, and computational modeling. **(A)** STN parcellation results. **(B)** Representation of white matter tracts between the STN and the cortex. **(C,D)** Lead locations with respect to the STN parcellation. **(E)** Patient-specific computational model of bilateral STN-DBS settings before and after right lead revision. The anatomical portion of the model was constructed from segmentation of high field imaging data (7T) with post-operative CT scans for lead localization. The STN volumes were then populated with biophysical multi-compartment neuron models that were perturbed with clinical DBS waveforms whose amplitudes were calculated from simulations of the tissue voltages induced through an anisotropic and inhomogeneous finite element model (FEM, COMSOL Multiphysics 5.4) of the electrode-tissue interfaces for this patient. The FEM was parameterized using diffusion-weighted imaging data from the patient. These models provided a quantitative estimate of the percentage of each neuronal pathway directly modulated by a clinical stimulation setting, with maximum possible activation of 100% for each pathway. Across all lead implants, the patient-specific models showed that stimulation of the motor STN was important to treat parkinsonian motor signs, while stronger activation of the associative and limbic territories resulted in the acute effects on mood.

To assess the location of the lead contacts and correlate these to the clinical outcome we used a combination of high-resolution 7T MRI and post-operative CT [for details see Duchin et al. (2018) and Figures 1A–D].

## Clinical and Imaging Methods

### Clinical Assessment

The motor effects of stimulation were assessed by calculating the sum of lateralized contralateral body Unified Parkinson’s Disease Rating Scale Part 3 (UPDRS III) subscore (left body tremor, rigidity, and bradykinesia). UPDRS III summed scores are also presented in Table 1.

### Scanning Protocol

In addition to the standard-of-care clinical imaging, the patient was also scanned with a 7T MRI (Magnetom 7 T Siemens, Erlangen, Germany) with SC72 gradients capable of 70 mT/m and a 200 T/m/s slew rate using a 32-element head array coil (Nova Medical, Inc., Burlington, MA, United States). The scanning protocol included: T1-weighted whole brain scan (0.6 mm^3^ isotropic, 6.5 min), T2-weighted coronal slab covering the whole STN and substantia nigra (0.4 mm × 0.4 mm × 1.0 mm, 6.5 min), and diffusion-weighted images, covering the whole brain (50 directions, *b*-value = 1500 s/mm^2^, 4 additional b0 volumes, 1.5 mm^3^ isotropic). The diffusion images were acquired twice, each with different phase encoding directions: anterior-posterior and posterior-anterior (acquisition time = 2 × 4.5 min). The patient was awake and on her regular medical regimen during the scanning session. Full scanning protocols are described in great detail in previous publications (Plantinga et al., 2014; Duchin et al., 2018; Patriat et al., 2018). High resolution, post-operative computed tomography data (0.3 mm × 0.3 mm × 0.6 mm, Siemens Biograph 64) were used 4 weeks after the first and the second (revision) DBS surgery.

### Image Processing and Analysis

Given the large variability in size, shape, and orientation of the STN (Duchin et al., 2018), manually segmenting the structure on the 7 T high-resolution images is more appropriate than utilizing a template. Therefore, three experts congruently performed the segmentation. Following our previously utilized techniques (Plantinga et al., 2018), probabilistic tractography was used as a primary tool to parcellate the STN into motor, associative, limbic, and “other” regions after performing motion, susceptibility and eddy current correction (FSL, RRID:SCR_002823). The post-operative CT and MRI images were non-linearly registered to determine the final location of the electrode with respect to the patient’s own anatomy (3D Slicer, RRID:SCR_005619; elastix, RRID:SCR_009619).

### Computational Modeling

The anatomical portion of the model was constructed from segmentation of high field imaging data (7T) with post-operative CT scans for lead localization. The STN volumes were then populated with biophysical multi-compartment neuron models that were perturbed with clinical DBS waveforms whose amplitudes were calculated from simulations of the tissue voltages induced through an anisotropic and inhomogeneous finite element model (FEM, COMSOL Multiphysics 5.4, RRID:SCR_014767) (Pena et al., 2018) of the electrode-tissue interfaces for this patient. T1-weighted anatomical images data were used to manually extract the brain from the cranial and extracranial anatomy (called lumped head tissue hereafter). Then the white matter, gray matter and cerebrospinal fluid brain tissue were segmented. The STN volumes were then populated with multi-compartment biophysical neuron models with realistic morphologies of dendrites, soma and axon and were perturbed with clinical DBS waveforms (Pulse width: 60 μs, Freq: 130 Hz) in NEURON using extracellular mechanism (NEURON, RRID:SCR_005393). The FEM was parameterized using diffusion-weighted imaging data from the patient. Together, these models provided a quantitative estimate of which neuronal populations were directly modulated by each clinical stimulation setting.

## Observations and Results

Using diffusion MRI data, a patient-specific tractography-based parcellation of the STN was performed (Figures 1A,B; Plantinga et al., 2018). This revealed a clear functional organization with partially overlapping zones, including a dorsal posterolateral motor region, a central associative region, and a smaller limbic region located anteromedially (Figures 1A–D). The left DBS lead was confirmed to be within the sensorimotor territory (Figure 1C). The right DBS lead was located in the anterior portion of the STN near the border between the limbic and associative territories (Figures 1C,D). Due to the stimulation-related adverse mood effects we chose to reposition the right lead posteriorly toward the sensorimotor region and the lead position was surgically revised. Following revision reconstruction of the right lead was confirmed to be within the STN sensorimotor territory (Figures 1C,D).

Motor benefit was measured by summing lateralized Unified Parkinson’s Disease Rating Scale Part 3 (UPDRS III) subscores as well as the UPDRS III total score and the relative degree of activation of the functional territories were modeled and correlated to the patient’s motor benefit and presence or absence of mood side effects (Table 1). Prior to DBS, the OFF medication left body UPDRS III subscore was 13 (Parkinson’s medications were held for at least 12 h prior to assessment). At 16 weeks, with stimulation ON at very low settings, due to low thresholds for adverse mood effects, the left body (Right STN) motor subscore was reduced to 9 (31% improvement). After the right lead was repositioned posteriorly into the motor territory, the left body motor subscore was reduced to 1 (92% improvement) without associated depression or anxiety. Note that this marked reduction included the lateralized left body scores only. The right body scores were reduced from 17 to 8 at 16 weeks (53% improvement) and to 2 after optimization post revision surgery (88% improvement). The additional benefit to the right body following revision were likely due to the change in contact (from 3−/C+ to 4−/C+) and small increase in amplitude (0.9 to 1.2 mA) of the left STN combined with potential ipsilateral benefit resulting from the revised right STN implant. The UPDRS III score was reduced from 48 to 31 at 16 weeks (35% improvement) and to 14 after optimization of the revised lead (71% improvement).

These clinical improvements correlated with a patient-specific computational model of the STN DBS settings that included an anisotropic and inhomogeneous finite element model (FEM, COMSOL Multiphysics 5.4) (Pena et al., 2018) coupled with a multi-compartment biophysical model of STN neurons (Miocinovic et al., 2006). This provided a quantitative estimate of the percentage of each modeled neuronal pathway (i.e., motor, associative, limbic, or other pathways within STN) modulated at each clinical stimulation setting. Before revision of the right STN lead settings, there was strong activation of both associative and limbic STN territories, with only weak activation of the motor territory (Figure 1E). In contrast, the clinical settings of the revised right lead as well as the left STN lead showed strong activation of motor STN neuronal populations, which correlated with greater motor benefit (Figure 1E).

## Discussion

This case report demonstrates the importance of lead location as a critical variable in determining STN DBS clinical outcomes. Using 7T MRI patient-specific STN parcellation, we provide a first-in-kind within-patient demonstration of superior motor outcomes with lead placement within the sensorimotor territory, while stimulation within associative and limbic regions provided less motor improvement and was associated with severe mood side effects. The significant improvement in motor benefit seen after repositioning the lead into the sensorimotor territory, is in agreement with previous studies that have suggested optimal motor benefit with stimulation of the dorsolateral motor STN (Wodarg et al., 2012). However, this result is in stark contrast to others who have argued that similar outcomes can be produced with stimulation anywhere within the STN (McClelland et al., 2005; Kasasbeh et al., 2013), or that the greatest motor improvements are seen in the most anterior electrode locations, closer to or in the associative territory (Welter et al., 2014).

This discrepancy may reflect the fact that these studies did not have the visualization tools that would allow for accurate determination of lead location with respect to individualized patient STN anatomy.

Previous studies were limited by: their use of low (McClelland et al., 2005) or intermediate (Kasasbeh et al., 2013; Welter et al., 2014) field MR imaging data making it difficult to accurately visualize the borders of the STN; imaging analyses that were not patient-specific (McClelland et al., 2005; Kasasbeh et al., 2013; Welter et al., 2014); utilizing AC-PC coordinates (McClelland et al., 2005) or Schaltenbrand atlas-based (Kasasbeh et al., 2013) lead localizations. Welter et al. (2014) used MR tractography, but employed a deformable atlas and theoretical STN subdivisions were extrapolated from tract tracings in non-human primates (Haynes and Haber, 2013; Welter et al., 2014), which does not take into account the significant between-patient variability of subcortical structures (Duchin et al., 2018; Plantinga et al., 2018).

Our finding of partial motor benefit with the lead placed more anteriorly was similarly reported in a recent study of STN DBS lead revisions in select patients with suboptimal motor benefit [as defined by inferiority to the patients suprathreshold ON medication motor scores (Nickl et al., 2019)]. This finding of partial motor improvement with lead location in the associative territory may help explain why some have argued that there is no difference between associative or motor territory stimulation (Welter et al., 2014). If we accept the baseline assumptions that: (1) there exist meaningful inter-individual anatomic variability of subcortical structures (Duchin et al., 2018; Plantinga et al., 2018), (2) imaging tools, until recently, have had limited ability to detect these differences, and (3) the STN functional territories include zones of considerable overlap (Haynes and Haber, 2013; Plantinga et al., 2018), then not only is the unresolved controversy over DBS lead location understandable, but we can also provide one additional explanation for why there has been such remarkably high variability of DBS outcomes within and across studies (Deuschl et al., 2006; Follett et al., 2010; Vitek et al., 2020).

In our patient, stimulation of the associative STN with spread into the limbic STN territory was likely responsible for her reversible disabling depression, transient hypomania, and feelings of euphoria before morphing into more persistent feelings of helplessness and depression. A remarkably similar case, the first reported in 1999, also observed the peak of negative mood effects 4 min after stimulation was turned on. However, the ability to determine the relative distribution of pathway activation responsible for the adverse effects was limited by the imaging and modeling technology of the time (Bejjani et al., 1999). Stimulation-induced hypomania is a well-recognized potential adverse mood effect of STN DBS (Mallet et al., 2007; Welter et al., 2014) that has been attributed to spread of stimulation into the putative limbic or associative STN territories. Our patient-specific imaging data (Figures 1A–D) and patient-specific computational models (Figure 1E) strongly support this hypothesis.

Reviewing the approach used for DBS lead placement we believe the low thresholds for stimulation induced paresthesia was the result of using a new device. With this device rather than abruptly scaling current amplitude up and down with a screener system to look for stimulation evoked muscle twitch, we assessed the patient for capsule effects associated with stimulation by disconnecting and reconnecting the stimulation cable. In retrospect this likely induced a capacitive discharge leading to the induction of intolerable paresthesia at low thresholds necessitating moving the lead from its initial implant site to a more anterior location.

While one could interpret the data based on a volume of tissue activated (VTA) approach, there is a growing number of studies showing that the VTAs are significantly less accurate than the pathway-specific approach adopted in this study (Gunalan et al., 2017; Howell et al., 2019). Additionally, the use of volumes is a misnomer when considering neuronal responses to stimulation, which have shown that electrical stimulation results in a sparse density map of neuronal activation (Histed et al., 2009; Xiao et al., 2018; Michelson et al., 2019).

This work is not without its limitations. Although this case report contains a cutting-edge imaging data set and analytics, e.g., 7T MRI patient-specific parcellation and modeling pathway activations, the work is based on a single patient. In support of the findings presented here, however, there are previous reports of mood changes during DBS that resolved when DBS was discontinued. The current study provides direct evidence in support of these studies while providing additional findings related to the relative effect of motor, associative and limbic pathway activations on clinical outcomes and side effects. Another potential limitation is movement artifact(s) associated with scanning movement disorder patients when using high-resolution MRI. To mitigate this problem scanning protocols were developed to minimize sensitivity to head motion [see (Duchin et al., 2018)]. A typical concern when scanning at 7T is an increased impact of susceptibility artifacts on diffusion data, especially in the phase encoding direction. To attenuate this issue, we followed the HCP (Glasser et al., 2013) scanning and preprocessing guidelines which include acquiring the diffusing “blip up” and “blip down” and using FSL topup/eddy current to minimize these distortions. Last while our imaging data were acquired with resolution higher than most clinical settings, partial volume of the diffusion imaging voxels may impact our ability to sharply define the borders between functional territories results given the relatively small volume of the STN.

## Conclusion

We are entering an era of rapid technological advance in the field of neuromodulation, with the development of powerful imaging technologies (Duchin et al., 2018; Plantinga et al., 2018), innovative segmented lead designs, tailorable programming capabilities, multiple current source devices, and predictive computational models of pathway activation (Gunalan et al., 2017; Pena et al., 2018). In this study, consistent with the hypothesis that DBS outcomes are critically dependent on lead location, we provide evidence that, in many cases, suboptimal DBS outcomes can be rationally explained, and corrected, on an individualized basis with only millimetric intra-target adjustments in DBS lead location (Nickl et al., 2019). Using ultra high field (7T) MRI, recently approved for clinical use, we present a tool with which we may be able to answer previously unresolved questions in the field, and by its very nature will bring us one step closer to patient-specific DBS.

## Data Availability Statement

The data that support the findings of this study are available on request from the corresponding author (JLV). The data are not publicly available due to them containing information that could compromise research participant privacy/consent.

## Ethics Statement

The studies involving human participants were reviewed and approved by the University of Minnesota’s IRB board. The patients/participants provided their written informed consent to participate in this study. Written informed consent was obtained from the individual(s) for the publication of any potentially identifiable images or data included in this article.

## Author Contributions

LES and RP contributed equally to this work. LES, RP, NH, and JLV designed the study, performed the majority of the analysis, and co-wrote the manuscript. MG, JK, and MDJ performed the computational modeling and participated in editing the manuscript. All authors contributed to the article and approved the submitted version.

## Conflict of Interest

LES has served as a consultant for Medtronic and Boston Scientific. RP is a consultant for Surgical Information Sciences, Inc. NH is a co-founder and shareholder in Surgical Information Sciences, Inc. JLV serves as a consultant for Medtronic, Boston Scientific, and Abbott, and serves on the scientific advisory board for Surgical Information Sciences, Inc. The remaining authors declare that the research was conducted in the absence of any commercial or financial relationships that could be construed as a potential conflict of interest.
